# Fast enrichment and detection of circulating tumor cells from large volumes of whole blood of breast cancer patients utilizing a functionalized bioaffinity CTC filtration membrane

**DOI:** 10.1002/ijc.70216

**Published:** 2025-10-27

**Authors:** Leonie F. Ott, Laura Keller, Nathan Bentley, Hümeyra Husseini‐Wüsthoff, René Werner, Marc Zinggeler, Jakoba Heidler, Parinaz Mossahebi Mohammadi, Cornelia Coith, Anne Pradines, Nikolas H. Stoecklein, Malte Löptien, Sven Peine, Maria Geffken, Corinna Güsmer, Mina Netkova‐Heintzen, Volkmar Müller, Elena Laakmann, Verena Thewes, Thomas M. Deutsch, Laura L. Michel, Andreas Schneeweiss, Andreas Trumpp, Jürgen Rühe, Thomas Brandtstetter, Sabine Riethdorf, Klaus Pantel

**Affiliations:** ^1^ Institute of Tumor Biology University Medical Center Hamburg‐Eppendorf Hamburg Germany; ^2^ Laboratoire de Biologie Medicale Oncologique Oncopole Claudius Regaud Toulouse France; ^3^ Laboratory for Chemistry and Physics of Interfaces, Department of Microsystems Engineering (IMTEK) University of Freiburg Freiburg im Breisgau Germany; ^4^ Department of Applied Medical Informatics University Medical Center Hamburg‐Eppendorf Hamburg Germany; ^5^ Department of Computational Neuroscience University Medical Center Hamburg‐Eppendorf Hamburg Germany; ^6^ Centre Suisse d'électronique et de Microtechnique SA (CSEM) Muttenz Switzerland; ^7^ Centre Suisse d'électronique et de Microtechnique SA (CSEM) Neuchatel Switzerland; ^8^ Department for General, Visceral and Pediatric Surgery University Hospital and Medical Faculty of the Heinrich Heine University Düsseldorf Düsseldorf Germany; ^9^ Department of Transfusion Medicine University Medical Center Hamburg‐Eppendorf Hamburg Germany; ^10^ Department of Gynecology University Medical Center Hamburg‐Eppendorf Hamburg Germany; ^11^ Division of Stem Cells and Cancer German Cancer Research Center (DKFZ) and DKFZ‐ZMBH Alliance Heidelberg Germany; ^12^ Department of Obstetrics and Gynecology Heidelberg University Hospital Heidelberg Germany; ^13^ National Center for Tumor Diseases, Heidelberg University Hospital German Cancer Research Center (DKFZ) Heidelberg Germany

**Keywords:** bioaffinity filter membrane, high‐volume samples, liquid biopsy, rapid CTC enrichment

## Abstract

Circulating tumor cells (CTCs) are valuable liquid biopsy analytes as they facilitate an in‐depth characterization of disseminated tumors by a simple blood draw. Most CTC assays can only process limited blood volumes, potentially hampering the detection of cells of very low frequency, such as CTCs. Here, we introduce a novel, functionalized nickel filtration membrane, facilitating rapid enrichment of CTCs from various volumes of blood up to 40 mL and leukapheresis products. We validated this assay with different cancer cell lines and compared the performance of our new assay with that of the FDA‐cleared CellSearch® system. Performing a comparative analysis of 20 blood samples from patients with metastatic breast cancer, CTCs were found in 19/20 patients (95%), combining the results of both CTC enrichment assays. CTC counts/7.5 mL of blood ranged from 0 to 7280 (CTC filtration membrane) and 0 to 17,190 (CellSearch®). Applying the clinically relevant cut‐off of ≥5 CTCs/7.5 mL of blood, 19/20 samples processed with our CTC filter were categorized identically to those analyzed with the CellSearch® system. Furthermore, the filtration time of 40 s for 7.5 mL of blood represents an important advantage of our system, compared to other systems. Importantly, we also demonstrated the feasibility of processing blood samples of up to 40 mL or 2 × 10^8^ nucleated blood cells in 2.5 min. Overall, our new system provides the advantage of performing ultra‐rapid enrichment of CTCs and upscaling the volume of blood that can be analyzed, which opens a new avenue for studies on CTC biology.

## Graphical abstract

What's new?

Circulating tumor cells (CTCs) are promising prognostic biomarkers for cancer. CTCs are sometimes very sparse; however, they present technical challenges for their enrichment from whole blood. Here, the authors tested the CTC enrichment capacity of a new nickel‐based filtration membrane and compared its efficacy to that of an FDA‐approved system. Analyses show that the novel CTC filter categorized samples identically to the system of comparison, but with a shorter filtration time and a superior ability to process high‐volume samples. Thus, the novel nickel membrane offers fast CTC enrichment and is a promising option in cases of minimal residual disease.

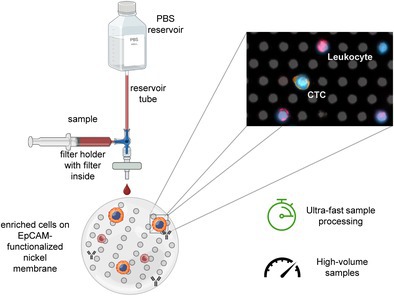

**Abbreviations**
BSA: bovine serum albumin
CHic: C,H insertion crosslinking reaction
CT: CellTracker
CTC: circulating tumor cell
ctDNA: circulating tumor DNA
ctmiRs: circulating tumor microRNAs
DAPI: 4′,6‐diamidino‐2‐phenylindole
DLA: diagnostic leukapheresis
DMEM: Dulbecco's modified Eagle medium
dPCR: digital PCR
EDTA: ethylenediaminetetraacetic acid
EGFR: Epidermal Growth Factor Receptor
EMT: epithelial‐to‐mesenchymal transition
EpCAM: epithelial cell adhesion molecule
FACS: fluorescence‐activated cell sorting
FCS: fetal calf serum
HD: healthy donor
HR: hormone receptor
HS: human serum
mBC: metastatic breast cancer
NMS: non‐maximum suppression
ODPA: octadecyl phosphonic acid
PCTE: polycarbonate track‐etched
PDMAA‐MABP: poly(dimethyl acrylamide‐co‐methacryloyloxy benzophenone)
PS‐Maz: 1‐(2‐(methacryloyloxy)ethyl) 3‐methyl 2‐diazomalonate
RPMI: Roswell Park Memorial Institute
RT: room temperature
tdEV: tumor‐derived extracellular vesicle
TNBC: triple negative breast cancer

## 1 INTRODUCTION

Due to the heterogeneity and complexity of the tumor disease, moving therapy for cancer patients toward a personalized approach for every patient is an urgent requirement.^1^ To find the best treatment option for every patient, an in‐depth characterization of the tumor is required. However, obtaining a tissue biopsy might not be feasible or appropriate sometimes, especially in the metastatic setting, and cannot be performed regularly due to the invasive nature of this procedure. Liquid biopsy, namely the characterization of the tumor lesions based on analytes in a blood draw, represents an attractive alternative.^2^

Circulating tumor cells (CTCs) are a particularly interesting analyte, and their number is negatively associated with the prognosis of cancer patients, as demonstrated by a plethora of clinical studies across multiple cancer entities and on a large number of patients.^3^ Furthermore, CTCs also offer the opportunity to analyze the transcriptome, proteome, epigenome, or genomic aberrations at the single‐cell level, thereby supporting unraveling tumor cell heterogeneity.^4^, ^5^ These possible applications also make them potential markers for tumor staging or disease monitoring.^6^ The usefulness of CTC phenotyping and enumeration was recently demonstrated in clinical studies, as these characteristics were successfully used to stratify patients into specific treatment arms.^7^, ^8^

However, even in the metastatic setting, CTCs can occur at very low concentrations,^9^ of sometimes less than one cell per ml of blood, and their enrichment from whole blood is therefore technically challenging. Most enrichment systems depend on distinctive traits of CTCs, which are either the expression of specific markers or their physical properties. For the enrichment and detection of CTCs from patients with metastatic colorectal, prostate, or breast cancer, the CellSearch® system, cleared by the FDA in 2004 for the enumeration of CTCs in these tumor entities, is still the gold standard. This system depends on the expression of the epithelial cell adhesion molecule (EpCAM), primarily expressed by epithelial cells but not mesenchymal ones, making its expression a unique feature of CTCs in the blood.^10^ Other enrichment systems, like the Parsortix® system, depend on the larger size of CTCs and their comparatively low deformability. Most of these systems are limited by the sample volume that can be processed. Frequently, the Parsortix® system is used for processing high‐volume samples as its maximum capacity is 40 mL.

Here, we combined an affinity‐ and size‐dependent enrichment of CTCs and developed a functionalized filtration system, offering rapid enrichment of CTCs from whole blood in less than 1 min. Based on previous work^11^ we developed a novel nickel membrane for CTC enrichment. This membrane offers lithographically defined, precise pore sizes and evenly distributed pore locations. We aimed to test the capacity of this membrane to enrich CTCs from clinical samples and compare its efficacy to that of the FDA‐cleared CellSearch® system. Furthermore, we investigated the feasibility of processing high‐volume samples, trying to bypass a limitation of various enrichment methods, as the usual sample size is limited to roughly 7.5–10 mL of blood. The CellSearch® system, for instance, is conceptualized to process up to 7.5 mL of whole blood and can process a maximum of 2 × 10^8^ cells per run.^12^, ^13^

This study introduces a new filtration‐ and bioaffinity‐based nickel membrane with the capacity to process high volumes of blood and high numbers of nucleated blood cells rapidly. We optimized its capacity to enrich tumor cells from whole blood in spiking experiments and validated the resulting CTC assay on clinical blood samples from an initial cohort of metastatic breast cancer patients. We provide encouraging proof‐of‐principle data demonstrating the feasibility of this new assay strategy.

## 2 METHODS

### 2.1 Cell culture

MCF‐7 (RRID:CVCL_0031), MDA‐MB‐231 (RRID:CVCL_0062), MDA‐MB‐468 (RRID:CVCL_0419), and Hs 578T (RRID:CVCL_0332) cell line cells were cultured in Dulbecco's modified Eagle medium (DMEM) at 37°C, 10% CO_2_, and SHP‐77 (RRID:CVCL_1693) cell line cells were cultured in Roswell Park Memorial Institute medium (RPMI) at 37°C, 5% CO_2_. All growth media were supplemented with 10% fetal calf serum (FCS) (v/v, PAA Laboratories) and 2 mM L‐glutamine (PAA Laboratories). All experiments were performed with mycoplasma‐free cells. All cell lines were authenticated within the last 3 years using Multiplex Cell Authentication by Multiplexion GmbH (Heidelberg, Germany). All cell lines were provided by our institutional pool of cell lines.

### 2.2 Cell staining and spiking

To test the efficacy of the EpCAM‐functionalized filtration membrane versus the CellSearch® system, MCF‐7, SHP‐77, and MDA‐MB‐231 cells were stained with CellTracker™ (CT) Orange CMRA, CellTracker Green CMFDA, or CellTracker™ Deep Red (Invitrogen™), respectively, in addition to serum‐free cell culture media (1:1000). After incubation for 30 min at 37°C, the cells were trypsinized for 3 min and collected in 1% BSA/PBS. One, ten, or hundred cells of each cell line were then spiked manually into 7.5 mL of healthy donor (HD) blood containing ethylenediaminetetraacetic acid (EDTA), which had been transferred into CellSave™ tubes. The enrichment was performed 1 h after the spiking, allowing the samples to be fixed by the CellSave™ fixative. All experiments were performed in triplicate. Cell diameters with and without CellTracker staining were assessed using the Vi‐CELL XR 2.06.3 (Beckman Coulter Inc.).

For the analysis of the cell‐enrichment based on the Epidermal Growth Factor Receptor (EGFR), MDA‐MB‐468, Hs 578T, and MDA‐MB‐231 cells were stained with CellTracker™ Orange CMRA, CellTracker Green CMFDA, or CellTracker™ Deep Red (Invitrogen™), respectively, in addition to serum‐free cell culture media (1:1000). After incubation for 30 min at 37°C, the cells were trypsinized for 3 min and collected in 1% BSA/PBS. Twenty‐five cells of each cell line were manually spiked into HD EDTA blood. The experiments were performed in triplicate.

### 2.3 Nickel membrane production

First, subsequent layers of silver (physical vapor deposition), Ti‐prime (spin coating), and the photoresist SPR 220‐7.0 (spin coating; Microresist Technology; ~20 μm) were deposited on a glass wafer. Using a photomask and UV‐light (dose E = 2.52 J/cm^2^), the desired pore pattern was illuminated on the photoresist. The photoresist is then developed with AZ 726 MIF (Microchemical GmbH), producing the pillar structures (~20 μm thick). The next step was to electroplate with Ni, followed by the removal of the photoresist and priming layers, resulting in a freestanding nickel membrane with a defined pattern and pore size. The membranes were brought to the appropriate size of the filter through a laser cutting step. The height of the deposited nickel was checked with a profilometer (target: 15 ± 4 μm) and the pore size under a microscope.

### 2.4 Nickel membrane coating and functionalization

The nickel membranes were cleaned in an ultrasonic bath in ethanol for 10 min, after which they were incubated overnight in octadecyl phosphonic acid (ODPA) dissolved in ethanol (1 mM). Following incubation, the samples were placed directly in an oven for 3 h at 100°C, then rinsed with ethanol as described in previous work.^14^, ^15^ A copolymer of styrene and 1‐(2‐(methacryloyloxy)ethyl) 3‐methyl 2‐diazomalonate (PS‐MAz) was coated onto the membranes through dip coating from ethyl acetate (50 mm/min), followed by simultaneous partial crosslinking and surface attachment through thermal activation (6 min, 160°C) and washing with ethyl acetate. An additional layer of poly(dimethyl acrylamide‐co‐methacryloyloxy benzophenone) (PDMAA‐MABP) was added through dip coating from PDMAA‐MABP dissolved in pure ethanol (99.8%) at a concentration of 5 mg/mL (50 mm/min), followed by partial crosslinking and attachment via UV activation (1 J/cm^2^, *λ* = 365 nm), and washing with ethanol. Streptavidin was drop‐coated on the membrane surface and allowed to dry overnight, followed by attachment via UV light (0.5 J/cm^2^, *λ* = 254 nm). The membranes were then washed with PBS‐0.1% Tween 20 and PBS. Using streptavidin‐biotin binding, further functionalization with biotinylated anti‐EpCAM antibodies can easily be achieved, enabling specific interaction with EpCAM^+^‐CTCs.

The ion‐track etched membranes used in some of the preliminary experiments were prepared in the same way as the nickel membranes, starting with the polymer deposition, as in this case, no ODP coating was necessary.

### 2.5 Patient cohort and blood samples

Twenty patients with metastatic Breast Cancer (mBC) of different molecular subtypes, either treated at the University Medical Center Heidelberg, Germany, or the University Medical Center Hamburg‐Eppendorf, Germany, were included in this study. Two 7.5 mL blood samples of each patient were collected in CellSave™ tubes (Menarini Silicon Biosystems). One sample was processed by the CellSearch® system (Menarini Silicon Biosystems), the current gold standard for the enrichment and enumeration of CTCs of patients with mBC. The other sample was processed using the bioaffinity CTC filtration membranes. The first 9 mL of flow‐throughs of four samples were collected and re‐analyzed by the CellSearch® system to detect cells that might not have been yet enriched.

### 2.6 Enrichment of epithelial cells from whole blood with the CellSearch® system

All samples were processed according to the manufacturer's protocol. Briefly, samples were drawn into CellSave™ tubes and processed within 96 h. They were spun down for 12 min at 800*g* without breaks. After the automated enrichment, using either the CTC or CXC kit, all cartridges were scanned at the CellTracks Analyzer II (Menarini Silicon Biosystems), and CTCs were identified by an experienced user (SR). If possible, samples were enriched by the CellSearch® system and the bioaffinity CTC filtration membrane on the same day.

### 2.7 Enrichment of epithelial cells from whole blood with the bioaffinity CTC filtration membrane

The CTC filtration system comprises a PBS container, a reservoir tube, and the filter, which is connected by a three‐way valve to the reservoir tube to facilitate the injection of the blood sample (graphical abstract). By adjusting the PBS volume, length of the reservoir tube, and distance from the filter to the PBS container, the pressure and flow velocity of the filtration process can be adjusted. For all experiments conducted in this study, a pressure of 34 mbar was used, which was experimentally determined in previous work.^11^

The bioaffinity CTC filtration membrane was inserted into an appropriate filter holder. After an initial washing step with 20 mL of 0.1% Tween/PBS, the filter, which had been functionalized with streptavidin, was incubated with 1 μg/mL biotinylated anti‐EpCAM‐antibody (R&D Systems) or 1 μg/mL biotinylated anti‐EGFR‐antibody (clone B1D8, Biotium) at RT for 1 h. Subsequently, the filter was washed with 20 mL of 0.1% Tween/PBS, and residual Tween was removed by washing the complete filtering system with 100 mL PBS. The blood sample was inserted through the three‐way valve and then filtered. The filtration was completed after 40 s. After completion of the filtration process, the sample was fixed by adding 10 mL of 70% ethanol, followed by 10 mL of 100% ethanol. For all spiking experiments as well as for the processing of the 7.5 mL patient samples, nickel membranes with a pore size of 5.5–6 μm were used.

Blood samples of higher volume (up to 40 mL) were processed using filter membranes of a larger diameter (47 mm) as well as a longer reservoir tube, and the filtration time was increased to 150 s.

### 2.8 Enrichment of epithelial cells from diagnostic leukapheresis products

The capacity of both enrichment systems to enrich CTCs from a diagnostic leukapheresis (DLA)^16^ product was tested. Since the maximum number of cells that can be processed by the CellSearch® system is 2 × 10^8^ cells, respective aliquots were used. To prepare these, the sample was centrifuged at 200*g* at RT without breaks. The cell number was adjusted to 1 × 10^8^ cells/mL using 5% Human Serum Albumin (HSA)/RPMI. Then, for CellSearch® analysis, the sample was filled with dilution buffer (Menarini Silicon Systems) to reach a final volume of 7.5 mL. For the filtration system, PBS was added to the sample to reach the same volume. Due to the high cell number, a filter with a 47 mm diameter was used. The nickel membrane had pores sized 7 μm and was functionalized with an anti‐EpCAM antibody as described before. The filtration time was 90 s.

### 2.9 Detection of recovered cells on the bioaffinity CTC filtration membrane by immunofluorescent staining

The fixed cells were immunofluorescently stained on the membrane. Unspecific binding sites were blocked by incubation with Dako antibody diluent with background‐reducing components (Agilent Technologies) for 15 min. Epithelial cells were detected by immunostaining with a cocktail including antibodies against EpCAM (clone VU1D9, 1:100 in PBS, Cell Signaling Technology) and two pan anti‐Keratin antibodies (clone AE1/AE3, 1:100; clone C11, 1:100, both Cell Signaling Technology). Contaminating leukocytes were identified by a cocktail of antibodies containing the exclusion markers CD45 (clone HI30, 1:200, Biolegend), CD66b (clone G10P5, 1:200, Biolegend), and CD16 (clone 3G8, 1:200, Biolegend). Nuclei were counterstained with 4′,6‐diamidino‐2‐phenylindole (DAPI) (1:500). The membranes were incubated with the antibody cocktail for 45 min at RT, washed three times with PBS, mounted with PBS, and sealed using FixoGum (Marabu). For microscopic imaging, the membranes were scanned at a Zeiss Axio Observer microscope at 50× magnification. Images were processed using Zeiss ZEN imaging software.

### 2.10 Statistics

Statistical analyses were performed using GraphPad Prism version 10 software. Statistical significance was reached with *p* ≤ .05 (*).

## 3 RESULTS

### 3.1 Polymer coating and functionalization of nickel membranes

**FIGURE 1:**
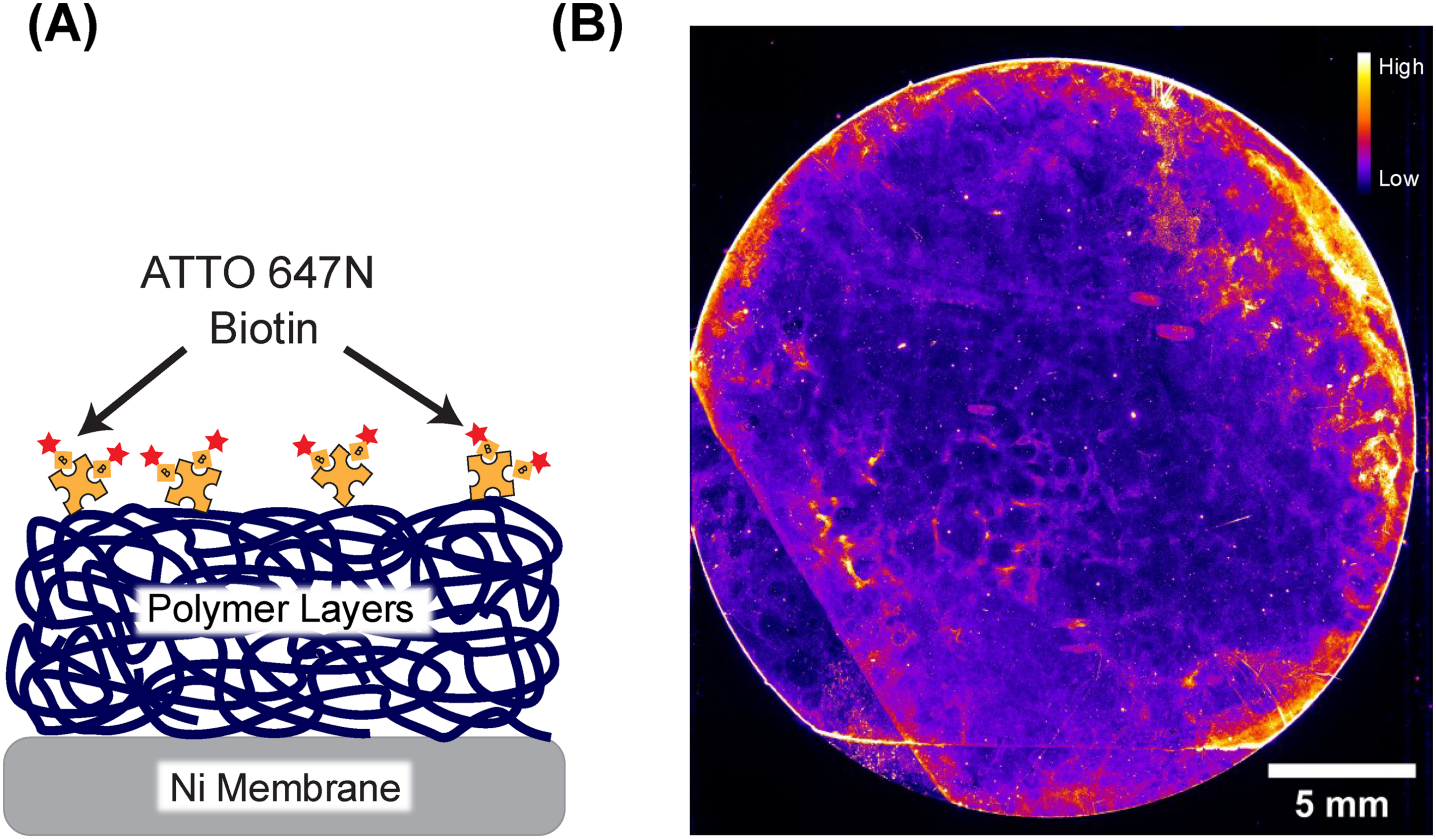
(A) Schematic depiction of the nickel membrane primed with ODP, coated with PS‐MABP and PDMAA‐MABP, and functionalized with streptavidin. For fluorescence readout, ATTO 647N biotin is attached to the streptavidin. (B) Fluorescence image (FLAIR reader, red channel, 100 ms) of coated and functionalized Ni membrane after incubation with ATTO 647N biotin. A high fluorescence signal can be seen with clear lines showing the edges of the polymer layers.^17^

The polymer coating, which has been designed to encapsulate the metallic substrate by a covalently attached thermally crosslinked polystyrene layer and to render them repellent against unspecific cell adhesion poly‐N,N′‐dimethyl acrylamide, obtained by photoactivation, was deposited on the patterned nickel membranes covered with a self‐assembled monolayer of ODP. The success of this process and the subsequent functionalization of the substrates with streptavidin were evaluated by imaging the binding of a fluorophore‐conjugated biotin (Figure 1A). The coated and functionalized membranes were incubated for 1 h with ATTO 647N Biotin dissolved in PBS (1 μg/mL). After incubation, the samples were washed with PBS‐0.1% Tween 20 and PBS, followed by fluorescence imaging using a FLAIR reader. An example of a resulting fluorescence image can be seen in Figure 1B. The fluorescence signals are well distributed on the membrane, indicating a good distribution of functional streptavidin binding sites.

### 3.2 Characteristics of cell lines used for EpCAM‐supported spiking experiments

Cancer cell lines with heterogeneous frequencies of EpCAM‐positive cells and of various cell sizes were chosen to reflect the heterogeneity of CTCs and test the ability of the bioaffinity CTC filtration membrane to enrich cells with different characteristics. Fluorescence‐activated cell sorting (FACS) analysis demonstrated that >99% of MCF‐7 (Figure S1A, Supporting Information) and SHP‐77 cells (Figure S1B) were EpCAM‐positive, but only 38% of MDA‐MB‐231 cells (Figure S1C) were identified as EpCAM‐positive. The intensity of the fluorescent anti‐EpCAM staining as well as the frequency of EpCAM‐positive cells was highest in MCF‐7 cells, intermediate in SHP‐77 cells, and lowest in MDA‐MB‐231 cells (Figure S1D).

The chosen cell lines also differed regarding their average cell size, as MCF‐7 had the largest average diameter of 17.2 μm, followed by MDA‐MB‐231 cells with an average diameter of 16.3 μm, and lastly SHP‐77 cells, which showed an average diameter of 12.7 μm. Notably, staining cells of all three cell lines with CellTracker™ dyes increased their diameter by 2.1–3.5 μm (Figure S1E).

### 3.3 Identification of the most suitable pore size

Naturally, the size of the pores of a membrane for size‐dependent CTC enrichment has a crucial impact on the efficacy of the enrichment process. Thus, MCF‐7 and SHP‐77 cells, which differ significantly (Mann–Whitney *U* test, *p* < .0001) in median cell size (Figure S1E), were used to identify the most suitable pore size. For that purpose, PCTE membranes were used. These membranes, unlike the nickel membranes used for the processing of the clinical samples, do not have evenly distributed pores, but the pores are randomly distributed due to a less complex manufacturing process. These membranes were used for initial experiments conducted to identify the best experimental parameters.

**FIGURE 2:**
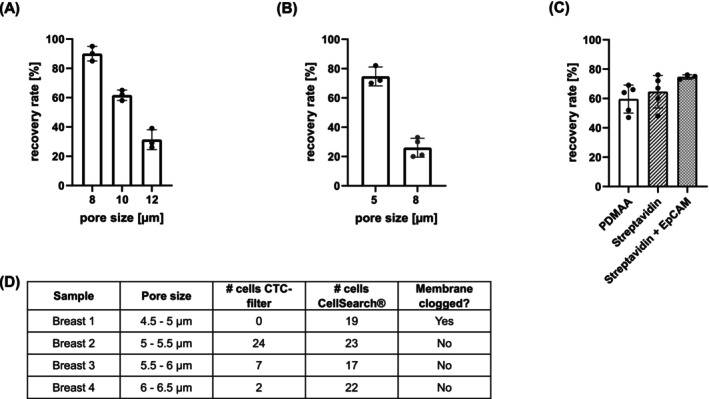
(A) Recovery rates of MCF‐7 cells using PCTE membranes with various pore sizes. 100 MCF‐7 cells were spiked into 2 mL of healthy donor blood. Experiments were performed in triplicate. (B) Recovery rates of SHP‐77 cells using PCTE membranes with two different pore sizes. 100 SHP‐77 cells were spiked into 2 mL of HD blood. Experiments were performed at least three times. (C) Improved recovery of SHP‐77 cells by membrane coating and EpCAM‐functionalization. Experiments were performed using nickel membranes with a 7 μm pore size and conducted at least three times.^17^ Error bars represent standard deviation (SD). (D) Overview of testing different pore sizes of nickel membranes for processing patient blood samples.

As Figure 2A demonstrates, the recovery rate of MCF‐7 cells dropped from 90 ± 5% achieved using membranes with pores of 8 μm size to 31.3 ± 6.8% when the pore size was increased to 12 μm. The experiment was repeated with SHP‐77 cells, whose average diameter is smaller than that of the MCF‐7 cells (12.7 and 17.2 μm, respectively). The SHP‐77 cells were spiked into HD blood using membranes with either 8 or 5 μm pores. Due to the smaller cell size, reducing the pore size further from 8 to 5 μm increased the recovery rate from 26 ± 6.5% to 74.7 ± 6.4% (Figure 2B).

The bioaffinity CTC filtration membrane provides the opportunity to combine size‐dependent CTC enrichment with marker‐based enrichment. EpCAM is an antigen that is expressed almost exclusively by epithelial cells, such as CTCs, but not mesenchymal cells, such as blood cells, and therefore is often used in systems depending on the specific expression of cell surface markers for the enrichment of epithelial CTCs. Therefore, EpCAM functionalization was chosen to increase the CTC capture efficacy in our clinical samples. Spiking experiments with SHP‐77 cells (Figure 2C) demonstrate that the addition of EpCAM‐dependent enrichment of the cells increased the recovery rates compared to non‐functionalized membranes, albeit to a nonsignificant degree (Kruskal–Wallis test, *p*‐value EpCAM vs. PDMAA = 0.0623). However, as CTCs are rare events (<1 CTC per 1 × 10^6^ WBC^18^), EpCAM functionalization was applied to process clinical samples to ensure the highest capture efficacy.

Due to the correlation between smaller pore sizes and improved recovery rates, nickel membranes with various pore sizes were tested initially on patient samples to identify the pore size providing the best recovery rate while not hampering the processing of the blood sample (results are summarized in Figure 2D). As similar blood volumes from patients with metastatic cancer tend to have more leukocytes than blood from healthy donors, clogging of the membranes with a pore size ≤5 μm was observed. Since with pore sizes between 5 and 6 μm (Figure 2D, samples breast 2 and 3) ≥5 CTCs/7.5 mL of blood were found in two cases with both devices, nickel membranes with pores sized between 5.5 and 6 μm were selected eventually for spiking experiments and the enrichment of CTCs from patient samples.

### 3.4 Evaluation of the recovery rate by spiking experiments

**FIGURE 3:**
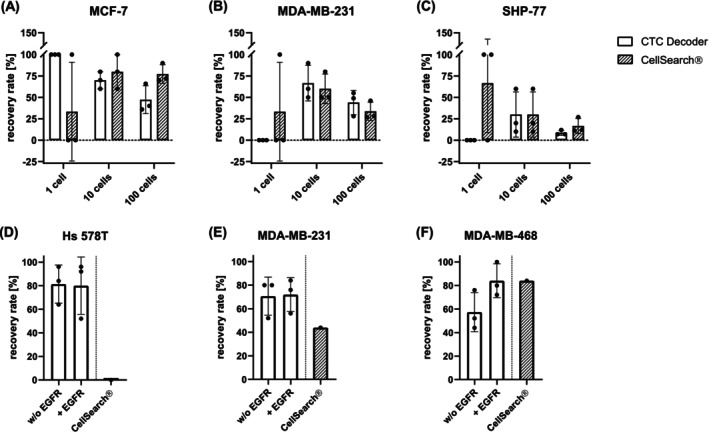
Recovery rates of spiking experiments using (A) MCF‐7, (B) MDA‐MB‐231, and (C) SHP‐77 cells. Of each cell line, either 1, 10, or 100 cells were spiked into 7.5 mL of HD blood. Experiments were performed in triplicate. Twenty‐five Hs 578T (D), MDA‐MB‐231 (E), and MDA‐MB‐468 (F) cells were spiked into 7.5 mL of HD blood to analyze the impact of membrane functionalization with an anti‐EGFR antibody on cell lines with intermediate to high EGFR and low to high EpCAM expression. All error bars represent SD.

The enrichment of CTCs from whole blood utilizing the bioaffinity CTC filtration membrane depends on the cell size and the abundance of cell‐surface markers, for example, EpCAM, whereas the CellSearch® system relies on EpCAM expression. The efficacy of the bioaffinity CTC filtration membrane to retrieve epithelial cells from whole blood was initially tested with spiking experiments, and the recovery rates were compared to those obtained with the CellSearch® system. For that purpose, different cell lines with specific features were tested (see Figure S1D,E) for EpCAM abundance and average diameters of the used cell lines. Of each cell line, (A) MCF‐7, (B) MDA‐MB‐231, (C) SHP‐77, 1, 10, and 100 cells were spiked into HD blood (Figure 3A–C). With both enrichment methods, MCF‐7 cells were most likely to be recovered (Figure 3A). Recovery rates of the bioaffinity CTC filtration membrane system versus the CellSearch® system for 1, 10, and 100 cells were as follows: 1 cell: 100% ± 0% vs. 33.3% ± 57.7%, 10 cells: 70% ± 10% vs. 80% ± 20%, 100 cells: 47.3% ± 16.3% vs. 77.3% ± 11.0%. These results demonstrate the bioaffinity CTC filtration membrane's performance compared to that of the CellSearch® system for the processing of samples with lower cell counts (1–10 cells/7.5 mL), but the latter is superior regarding the enrichment of higher cell numbers.

Similarly, the recovery rates of MDA‐MB‐231 cells were comparable between the two enrichment methods (Figure 3B). A single spiked MDA‐MB‐231 cell was recovered once with the CellSearch® (33.3 ± 57.7%) system but not with the bioaffinity CTC filtration membrane. Spiking 10 MDA‐MB‐231 cells, 66.7 ± 20.8% were recovered using the bioaffinity CTC filtration membrane and 60.0 ± 17.3% using the CellSearch® system. Of 100 spiked MDA‐MB‐231 cells, 44.0 ± 14.7% were recovered with the bioaffinity CTC filtration membrane, and 33.7 ± 10.7% were recovered with the CellSearch® system.

The recovery of SHP‐77 cells from EDTA blood was the least efficient using any method (Figure 3C). Recovery rates of the bioaffinity CTC filtration membrane system versus the CellSearch® system for 1, 10, and 100 SHP‐77 cells were as follows: 1 cell: 0% vs. 66.7% ± 57.7%, 10 cells: 30.0% ± 26.5% vs. 30.0% ± 26.5%, 100 cells: 8.7% ± 2.9% vs. 16.7% ± 9.0%.

However, albeit rather rarely observed in breast cancer, not all CTCs show EpCAM expression, either because they might have undergone epithelial‐to‐mesenchymal transition (EMT) or because of their tissue origin. Thus, we tested EGFR as an alternative marker to aid in the capture of EpCAM low or even negative cells. Twenty‐five MDA‐MB‐468, MDA‐MB‐231, and Hs 578T cell line cells were spiked into HD donor blood and processed with 5.5–6 μm pore size nickel membranes, either functionalized with or without an anti‐EGFR antibody. Results show that the recovery rate of neither Hs 578T nor MDA‐MB‐231 cells, which both show intermediate EGFR cell surface levels (Figure S2), was affected by the functionalization with an anti‐EGFR antibody, resulting in recovery rates of approximately 80% and 70%, respectively (Figure 3D,E, respectively). However, using EGFR‐high MDA‐MB‐468 cells, the recovery rate was improved from 57.3% ± 16.7% to 84% ± 14.4% by providing bioaffinity‐based enrichment (Figure 3F), albeit to a nonsignificant degree (Mann–Whitney test *p* = .2). A comparative CellSearch® experiment resulted in 0% recovered EpCAM‐negative Hs 578T cells, 44% of recovered MDA‐MB‐231 cells, which have low EpCAM expression, and 84% recovered MDA‐MB‐468 cells, which have a high EpCAM expression (Figure S2).

### 3.5 Recovery rates of CTCs from clinical blood samples: Bioaffinity CTC filtration membrane versus CellSearch®

Since the spiking experiments using membranes functionalized with an anti‐EpCAM antibody demonstrated the comparability of the bioaffinity CTC filtration membrane with the CellSearch® system, both approaches were used for processing patient samples.

**FIGURE 4:**
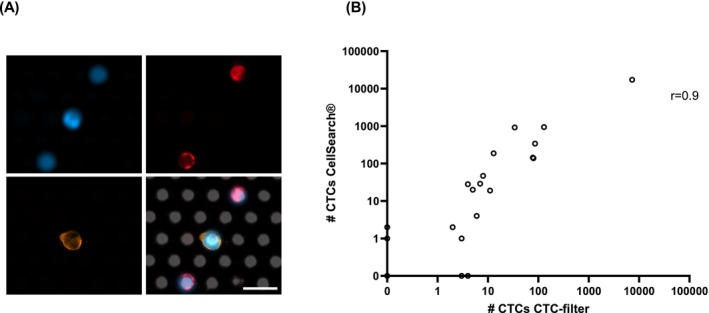
(A) Exemplary picture of a CTC on the bioaffinity CTC filtration membrane. Nuclei were stained with DAPI (blue). Leukocytes were identified by CD45, CD16, and CD66b staining (all red), and CTCs by staining against pan‐Keratin and EpCAM (both orange). The scale bar represents 20 μm. (B) Graphical illustration of the correlation between CTC counts obtained with the bioaffinity CTC filtration membrane vs. CellSearch®. Spearman coefficient *r* = 0.9.

Twenty blood samples from 18 patients with metastatic breast cancer of different molecular subtypes (hormone‐receptor positive [HR] and triple‐negative (TNBC)) were analyzed in parallel using both enrichment methods, and CTCs were identified as nucleated, EpCAM‐ and keratin‐positive cells that were negative for leukocyte markers. In line with the spiking experiments, the enrichment of CTCs was comparable between the two methods (results are summarized in Table 1). An exemplary picture of a CTC identified on the bioaffinity CTC filtration membrane is shown in Figure 4A. Applying the threshold of ≥5 morphologically intact CTCs/7.5 mL of blood, which is of prognostic relevance using the CellSearch® system, 19/20 samples were categorized identically. In one case, 11 morphologically intact cells were identified using the bioaffinity CTC‐filtration membrane, but only three morphologically intact cells were found with the CellSearch® system, resulting in differential categorization. Using the bioaffinity CTC filtration membrane, the number of intact cells was lower in 10/20 cases compared to the results obtained with the CellSearch® system, whereas higher CTC numbers were detected in 7/20 cases. In one sample, no intact CTC was detected by either system, and in 2/20 samples, the same number of CTCs was detected with both methods. After enrichment with the bioaffinity filtration membrane, CTCs were found in 17/20 cases, whereas 16/20 cases were CTC‐positive using the CellSearch® system. However, in total, more intact CTCs were found with the CellSearch® system than with the bioaffinity CTC filtration membrane (mean 848 vs. 386; median 4 vs. 6, respectively). Additionally, the total number of non‐intact CTCs found with the CellSearch® system was higher than that obtained with the bioaffinity CTC filtration membrane (Table 1). CTC clusters, defined as at least two attached CTCs, were found in 3/20 samples using the bioaffinity CTC filtration membrane and in 1/20 samples using the CellSearch® system.

**TABLE 1:** Summarized data of CTC counts enriched with the bioaffinity filtration membrane vs. CellSearch®.

Sample ID	Molecular subtype	CTC‐filter			CellSearch®			CellSearch® flow through	Cell‐Search® Test Kit
		Total	Intact	Non‐intact	Total	Intact	Non‐intact		
#1	HR+	0	0	0	1	1	0	N/A	CTC
#2	HR+	2	2	0	2	2	0	N/A	CTC
#3	HR+	3	2	1	0	0	0	N/A	CTC
#4	HR+	13	11	2	187	86	101	N/A	CTC
#5	TNBC	4	4	0	28	3	25	N/A	CXC
#6	HR+	3	3	0	1	0	1	N/A	CTC
#7	HR+	86^a^	78	8	341	278	63	N/A	CTC
#8	HR+	7280^a^	7280^b^	0	17,190^a^	15,300^b^	1890^b^	N/A	CTC
#9	TNBC	5	5	0	20	5	15	N/A	CXC
#10	HR+	8	8	0	47	27	20	N/A	CTC
#11	HR+	7	7	0	29	22	7	N/A	CTC
#12	HR+	34	34	0	920	320^b^	600^b^	N/A	CTC
#13	HR+	78	71	7	144	99	45	N/A	CTC
#14	HR+	6	2	4	4	1	3	N/A	CTC
#15	TNBC	11	11	0	19	3	16	N/A	CXC
#16	HR+	1	0	1	0	0	0	1	CTC
#17	TNBC	80	80	0	139	79	60	31 intact, 73 non‐intact (total 105)	CXC
#18	HR+	130^a^	127	3	940	740^b^	200^b^	N/A	CTC
#19	HR+	4	2	2	0	0	0	0	CTC
#20	HR+	0	0	0	2	2	0	0	CTC

^a^ CTC clusters detected.

^b^ CTC counts were extrapolated.

From four samples enriched with the bioaffinity CTC filtration membrane, the first ~9 mL of flow‐through was collected and resubstituted for analysis by the CellSearch® system, to analyze how many cells were not enriched by the bioaffinity CTC filtration membrane. However, CTCs were detected only in two.

Graphical analysis of the comparative enrichment shows a good correlation between the bioaffinity CTC filtration membrane and the CellSearch® system, with a Spearman coefficient of 0.9 (Figure 4B).

Frequently, CTCs are used for various downstream analyses after enrichment. For that purpose, a low leukocyte background might be preferred to increase the sensitivity of the analysis. To determine the capacity of each method to deplete leukocytes from the samples, nuclei on the bioaffinity CTC filtration membranes and the CellSearch® cartridges were automatically detected and counted using StarDist^19^, ^20^ (Table S2). The comparison reveals that the CellSearch®‐enriched samples contained lower numbers of leukocytes than the samples enriched with the bioaffinity CTC filtration membrane (median 22,412 vs. 651,744; mean 37,327 vs. 623,208, respectively).

### 3.6 Processing high‐volume samples

The bioaffinity CTC filtration membrane system not only provides flexibility regarding the used pore size and the selected capture antibody but also facilitates the processing of high‐volume samples. To filter up to 40 mL of whole blood, larger‐sized nickel membranes (47 mm) were used. To test the capacity of the system to retrieve CTCs from high‐volume blood samples, 10 MCF‐7 cells were spiked into 40 mL of HD blood.

Despite a low recovery rate of only 30 ± 0% obtained from MCF‐7 cells spiked into 40 mL of HD blood (*n* = 2), the technical feasibility of processing high‐volume blood samples from mBC patients was tested on two patient samples. For that purpose, membranes with different pore sizes were also tested to potentially find a more suitable experimental setting.

As the results summarized in Table 2 depict, the processing of high‐volume blood samples and the enrichment of CTCs from them is feasible. The results from sample #1 indicate a linearity between the number of CTCs and the blood volume analyzed. Sample #2 demonstrates that increasing the analyzed volume of blood indeed increases the chance of finding CTCs in cases where no CTCs were found in a typical 7.5 mL blood draw. Here, two intact cells were enriched from 30 mL of EDTA blood with the bioaffinity CTC filtration membrane, whereas using the CellSearch® system, no CTC was detected in only 7.5 mL of blood. Thus, increasing the blood volume analyzed can be beneficial to increase not only the number of enriched CTCs, facilitating better disease characterization, but also the likelihood of finding CTCs in individuals with low‐tumor burden.

**TABLE 2:** CTC numbers after enrichment of high‐volume blood samples processed with the CTC filtration membrane vs. CellSearch®.

Sample	Pore size (μm)	Volume CTC‐filter	# Cells CTC‐filter			Volume CellSearch®	# Cells CellSearch®		
			Total	Intact	Non‐intact		Total	Intact	Non‐intact
1	7	32 mL	11	9	2	7.5 mL	5	2	3
2	5.5–6	30 mL	3	2	1	7.5 mL	0	0	0

To demonstrate the broad utility of our filtration system, we also processed a DLA sample and compared the results with those of the CellSearch® system. Using 2 × 10^8^ white blood cells for each system, 12 morphologically intact cells were recovered by the CellSearch® system, and 10 using the filtration membrane. Interestingly, apoptotic cells and tumor‐derived extracellular vesicles (tdEVs), the clinical importance of which has been demonstrated recently^21^ were found in the samples enriched with both methods, demonstrating the filtration membranes' usability for processing DLA samples as well.

### 3.7 Molecular characterization of enriched tumor cells by dPCR

Characterizing CTCs molecularly may provide therapeutically relevant information about the tumor. Thus, we used dPCR to test the compatibility of CTCs captured with bioaffinity filtration membranes with genomic downstream analysis. For that purpose, we aimed to detect the *PI3KCA* E545K mutation in MCF‐7 cells that were spiked into HD blood. The lack of signal in the water control (Figure S3A) and the detection of a specific signal in the positive control (Figure S3B) demonstrated specificity of the analysis. The threshold for positivity was determined using two HD blood samples, in which only 5.8 [95% CI: 0–11.5] (Figure S3C) and 4.0 [95% CI: −1.15–6.9] (Figure S3D) mutant copies were detected, respectively. Based on a 95% confidence interval, the *PI3KCA* E545K mutation was detected in both spiking samples analyzed, with 52.9 [95% CI: 35.7–70.2] copies detected in one sample of 114 spiked MCF‐7 cells among 72,657 *PIK3CA* WT [95% CI: 71,990–73,336] copies (Figure S3E) and 94.3 [95% CI: 71.3–117.3] copies detected in a sample with 182 MCF‐7 cells among 156,055 *PIK3CA* WT [95% CI: 155,020–157,090] copies (Figure S3F), indicating nearly linearity in this assay and good correspondence with the amount of tumor cells per sample. Summarized, these results demonstrate that genomic downstream analysis by dPCR is feasible on tumor cells enriched from blood samples, which were processed with our bioaffinity filtration membrane.

## 4 DISCUSSION

Here, we introduced a new filtration membrane for the enrichment of CTCs. Our system provides several advantages over commonly used CTC enrichment systems, such as an outstandingly short filtration time of less than a minute for 7.5 mL of blood and flexibility regarding the functionalization with an antibody against a target of choice.

By that, we hope to overcome the hurdles of other enrichment systems, which are commonly limited by sample capacity^22^, ^23^ or by processing time. Furthermore, by small adjustments, the system can also be used to process high‐volume samples of at least up to 40 mL of blood or up to 2 × 10^8^ nucleated blood cells, making it also an interesting option for CTC enrichment in the context of minimal residual disease.^24^, ^25^

Since CTCs occur at very low frequencies in individuals with low tumor burden, increasing the sample volume for analysis has gained increasing interest. A method that gained popularity in increasing CTC numbers is DLA.^12^, ^13^ By continuous filtration of blood and thereby enrichment of CTCs from up to 2.5 L of blood per patient, DLA products contain a high density of cells of approximately 72 × 10^6^/mL.^12^ Thus, systems that can process high‐volume samples rapidly are required. Not only did the filtration membrane recover a comparable number of morphologically intact cells as the CellSearch® system (10 vs. 12) and likewise enriched apoptotic cells and tdEVs, but it did so in a fraction of the time, as the filtration only took 90 s. This demonstrates the suitability of our filtration membrane to process complex samples with a high number of cells in an extremely short amount of time.

The rapid processing time of our system and the possibility of processing high‐volume samples represent major advantages over comparable systems. Several systems, combining at least two modalities for CTC enrichment, such as our bioaffinity CTC filtration membrane, have a high processing time of only several milliliters per hour.^26^ Furthermore, not all systems were tested on clinical samples or compared to commercial CTC enrichment systems. Another benefit of our filtration membrane is that it can process whole blood and does not need any preparation, such as red blood cell lysis or sample dilution, before enrichment.

Utilizing spiking experiments, the bioaffinity CTC filtration membrane was successfully compared to the CellSearch® system, representing the current gold standard for the enrichment of CTCs from patients with mBC. Testing our membranes on a proof‐of‐principle cohort of 20 patients with mBC demonstrated that the system provides results comparable to the CellSearch® system, also on clinical samples, resulting in the same prognostic categorization (≥5 morphologically intact CTCs/7.5 mL of blood) of samples in 95% of cases analyzed. Therefore, it might represent an interesting alternative to the EpCAM‐dependent CellSearch® system.

However, this study also bears some limitations. First, despite resulting in CTC counts with comparable prognostic categorization as the CellSearch® system, the total number of CTCs, especially of non‐intact cells, was considerably lower using the bioaffinity CTC filtration membrane, indicating that maybe only robust and intact CTCs can be captured, but not more fragile, non‐intact, or very small CTCs. This could be disadvantageous for certain studies as the prognostic relevance and inverse correlation with overall survival (OS) and progression‐free survival (PFS) of apoptotic CTCs have already been shown in the clinical setting.^27^ Furthermore, we focused on the enrichment of CTCs from mBC patients, as the CellSearch® system is an FDA‐approved standard for CTCs of this entity. However, CTCs from breast cancer have, on average, a larger diameter than leukocytes, facilitating their size‐dependent enrichment. Other entities, such as bladder or colorectal cancer, tend to have smaller CTCs, which might make it difficult to separate them from leukocytes by filtration.^28^ We used a small cell lung cancer cell line in our study, with a comparatively small cell diameter, to test the capacity of our membranes to also enrich smaller cells. Indeed, we yielded the lowest recovery rates for this cell line, perhaps attributable to its size. This issue might be tackled by reducing the pore size of the filtration membranes.

We assume that by adapting our bioaffinity CTC filtration membrane with antibodies against other CTC antigens, including markers for epithelial‐to‐mesenchymal transition EMT,^29^, ^30^ we might also enrich CTCs, which might not be caught by the CellSearch® system, probably due to a lack of EpCAM expression on the cell surface. To test this hypothesis, we also used membranes functionalized with an anti‐EGFR antibody. Spiking three cell lines with variable EGFR and EpCAM levels, we only observed a nonsignificant increase in the recovery of strongly EGFR‐positive MDA‐MB‐468 cells. This result and the rather small increase in the number of SHP‐77 cells recovered by the functionalization with an anti‐EpCAM antibody led to the assumption that the recovery efficacy of our filtration membrane is mainly dependent on the pore size and geometry. Comparison to the CellSearch® system, using cell line cells with EGFR but various EpCAM expressions, demonstrated the strong dependency of this system on EpCAM expression and the superiority of our bioaffinity filtration membrane in catching EpCAM‐negative cells. We still opted to functionalize our membranes with an anti‐EpCAM antibody to maximize the number of CTCs that can be enriched, since recovery rates were nonsignificantly improved by functionalizing the membrane with antibodies against surface‐specific antigens such as EpCAM and EGFR, using either SHP‐77 and MDA‐MB‐468 cells, respectively, as every single CTC can carry relevant information and represent a tumor's heterogeneity.

However, whether all the CTCs captured by the functionalized filtration membrane were EpCAM‐positive or whether EpCAM‐negative CTCs were also enriched had not been tested, as we used an antibody cocktail with EpCAM and keratin antibodies in the same color, hampering the differentiation of EpCAM‐/Keratin+ and EpCAM+/Keratin+ CTCs. Finally, we still suffer from a considerable leukocyte background, which might impede downstream analyses of the cells of interest. Therefore, improving the membrane, for example, the pore size, pore arrangement, antibody coating, or pore density, would be required.

Nonetheless, we were able to detect cancer cell‐specific mutations from MCF‐7 cells spiked into HD donor blood, demonstrating the feasibility of genomic analysis despite the comparably high leukocyte background, if a sensitive analysis method is chosen. These results imply that using our bioaffinity filtration membrane facilitates molecular characterization of the captured CTCs. Albeit not on a single‐cell level yet, bulk mutation analysis, as performed in this study, could provide clinically or therapeutically relevant information beyond the mere enumeration of CTCs, feasible with the bioaffinity filtration membrane.

## 5 CONCLUSION

Our study introduces a novel functionalized CTC filtration membrane, providing an adaptable platform for extremely fast enrichment of CTCs from blood samples of up to 40 mL volume, as well as for high numbers of cells enriched from a DLA product within a few minutes. The very short processing time and the flexibility regarding the sample volume, the pore size, and the possibility of functionalizing the membrane with an antibody of choice, as well as its compatibility with genomic downstream analysis of the captured CTCs, make this system attractive for future applications beyond breast cancer.

## AUTHOR CONTRIBUTIONS

**Leonie F. Ott:** Validation; investigation; project administration; visualization; formal analysis; writing – original draft; writing – review and editing; methodology. **Laura Keller:** Validation; investigation; visualization; formal analysis; project administration; writing – original draft; writing – review and editing; methodology; resources. **Nathan Bentley:** Validation; investigation; visualization; project administration; writing – original draft; writing – review and editing; formal analysis. **Hümeyra Husseini‐Wüsthoff:** Validation; writing – original draft; methodology; writing – review and editing; formal analysis; resources. **René Werner:** Methodology; supervision; writing – review and editing; funding acquisition; project administration; resources. **Marc Zinggeler:** Methodology; investigation; writing – review and editing. **Jakoba Heidler:** Methodology; investigation; writing – review and editing. **Parinaz Mossahebi Mohammadi:** Validation; investigation; visualization; project administration; writing – review and editing; formal analysis. **Cornelia Coith:** Investigation; writing – review and editing. **Anne Pradines:** Investigation; writing – review and editing. **Nikolas H. Stoecklein:** Investigation; writing – review and editing. **Malte Löptien:** Investigation; writing – review and editing. **Sven Peine:** Investigation; writing – review and editing; resources. **Maria Geffken:** Investigation; writing – review and editing; resources. **Corinna Güsmer:** Investigation; writing – review and editing; resources. **Mina Netkova‐Heintzen:** Investigation; writing – review and editing; resources. **Volkmar Müller:** Investigation; funding acquisition; writing – review and editing; resources. **Elena Laakmann:** Investigation; writing – review and editing; resources. **Verena Thewes:** Investigation; writing – review and editing; resources. **Thomas M. Deutsch:** Investigation; writing – review and editing; resources. **Laura L. Michel:** Investigation; writing – review and editing; resources. **Andreas Schneeweiss:** Investigation; writing – review and editing; resources. **Andreas Trumpp:** Investigation; writing – review and editing; resources. **Jürgen Rühe:** Conceptualization; resources; supervision; funding acquisition; methodology; writing – review and editing; project administration. **Thomas Brandtstetter:** Conceptualization; resources; supervision; funding acquisition; methodology; writing – review and editing; project administration. **Sabine Riethdorf:** Conceptualization; resources; supervision; writing – review and editing; validation; funding acquisition; project administration. **Klaus Pantel:** Conceptualization; resources; supervision; funding acquisition; writing – review and editing; project administration.

## CONFLICT OF INTEREST STATEMENT

The authors declare no conflicts of interest.

## ETHICS STATEMENT

CTC‐analyses were approved by the ethical committee of the University of Heidelberg (case numbers S295/2009 and S‐164/2017; NCT05652569), the University of Mannheim (2010‐024238‐46), and the Ethikkommission der Ärztekammer Hamburg (5392‐3704‐BO). All patients provided their written consent.

## Supporting information


**Data S1.** Supporting Information.

## Data Availability

The data that support the findings of this study are available from the corresponding author upon reasonable request.
