# Supplementary material for: Fast enrichment and detection of circulating tumor cells from large volumes of whole blood of breast cancer patients utilizing a functionalized bioaffinity CTC filtration membrane
Source: Int J Cancer. 2025 Oct 27;158(6):1713–25. doi: 10.1002/ijc.70216 (PMC12811197; doi:10.1002/ijc.70216)
Supplement: Supplementary file 1 — Data S1. Supporting Information. [file IJC-158-1713-s001.pdf]

Fast enrichment and detection of circulating tumor cells from large volumes of whole blood of breast cancer patients utilizing a functionalized bioaffinity CTC filtration membrane

Leonie F. Ott, Laura Keller, Nathan Bentley, Hümeyra Husseini-Wüsthoff, Rene Werner, Marc Zinggeler, Jakoba Heidler, Parinaz Mossahebi Mohammadi, Cornelia Coith, Anne Pradines, Nikolas H. Stoecklein, Malte Löptien, Sven Peine, Maria Geffken, Corinna Güsmer, Mina Netkova-Heintzen, Volkmar Müller, Elena Laakmann, Verena Thewes, Thomas M. Deutsch, Laura L. Michel, Andreas Schneeweiss, Andreas Trumpp, Jürgen Rühe, Thomas Brandtstetter, Sabine Riethdorf, Klaus Pantel

Table of contents

Supplementary Material and Methods.....2
Supplementary Tables.....6
Table S1.....6
Table S2.....7
Supplementary Figures.....8
Figure S1.....8
Figure S2.....9
Figure S3.....10
Supplementary References.....11

Supplementary Methods

EpCAM analysis by fluorescence-activated cell sorting analysis

Cell culture cells were harvested, and the cell number was adjusted to $1x10^6$ cells/ml in PBS. A cell viability dye was added to the samples (Zombie NIR dye™, 1:500, Biolegend), followed by incubation for 15 minutes at room temperature (RT). Cells were washed with 2 ml of 0.5% bovine serum albumin (BSA)/PBS and centrifuged at 300x g for 10 minutes. Then, cells were incubated with an anti-EpCAM antibody (REA764, 1:50, Biolegend) or an Isotype control (mouse IgG_1, 1:50, Santa Cruz Biotechnology) for 10 minutes at 4 °C. The cells were washed again, and the pellet was resuspended in 500 µl of 0.5% BSA/PBS. The samples were filtered through a 40 µm cell sieve, and the EpCAM expression on live cells was assessed on an Agilent NovoCyte Quanteon. In brief, the cell population was gated in an FSC-H vs SSC-H blot, and single cells were identified employing an FSC-A vs FSC-H blot. Unstained cells and isotype controls were used to discriminate between positive and negative populations for the viability dye and EpCAM staining. For that, FSC-H vs either NIR-H or FITC-H blots were used. Only cells negative for the viability dye (=intact cells) were included in the analysis of EpCAM expression. Without further processing, the plots were saved as EMF files.

Anti-EGFR and EpCAM immunostaining

Cytospins of MCF-7, MDA-MB-231, Hs 578T, and MDA-MB-468 cells, spiked into the buffy coat of HD blood, were produced by a three-minute centrifugation at 244x g. After drying, the cells were fixed with 4% PFA for 15 min at RT, followed by three three-

minute washing steps with PBS. Subsequently, cells were permeabilized using 0.2% Triton X-100 in PBS for 10 minutes. Cells were washed as described before. Unspecific binding sides were blocked using 10% human serum (HS) (v/v, Sigma Aldrich). The cells were incubated with the primary antibody, anti-EGFR (B1D8, 1:50 in HS, Biotium) for 60 minutes, washed again three times with PBS, followed by an incubation with the secondary antibody (Alexa Fluor 555 goat anti-mouse, 1:200 in Dako antibody diluent with background-reducing components, Invitrogen) for 45 minutes. EpCAM was stained using a directly-labeled antibody (VU1D9, 1:200 in Dako antibody diluent with background-reducing components, Cell Signaling Technology). Leukocytes were excluded by anti-CD45 staining (HI30, 1:200, Biolegend), and nuclei were counterstained using DAPI (1:500) for 45 minutes. After three final washing steps, the slides were mounted using Fluoromount-G (Invitrogen) and analyzed at a Zeiss Axio Observer microscope at 200x magnification. Images were processed using Zeiss ZEN imaging software.

Image-based cell detection and automated leukocyte enumeration from CellSearch® data

StarDist was applied to both the cartridge images from the CellSearch® system and the bioaffinity CTC filtration membrane images. [1,2] The images of the scanned bioaffinity CTC filtration membranes were screened manually to detect CTCs. A cell was considered a CTC if it was positive for the inclusion markers, had an intact nucleus, and did not show any signal in the channel of the exclusion markers. The number of leukocytes was determined by an automated algorithm, which includes a segmentation component called StarDist.[3] StarDist is a method for detecting cells or nuclei in microscopy images that utilizes star-convex shape assumptions. For the

enumeration of leukocytes, the pre-trained model 2D_versatile_fluo (pre-trained on microscopy images) was used. The non-maximum suppression (NMS) parameters were set as follows: prob_thresh=0.6, which controls the number of detected objects and reduces the likelihood of false positives, and nms_thresh=0.2, which controls the overlap of detected objects.

For the CellSearch® system, the detection was performed on the DAPI channel across 175 cartridge images per sample, and the detected objects were summed. In contrast, due to the larger dimensions of the bioaffinity CTC filtration membrane images (approximately 23120 × 22480 pixels) compared to the CellSearch® cartridge images (1384 × 1036 pixels), each image was partitioned into 100 patches. StarDist was then applied to each patch, and the detected objects were totaled. It must be mentioned in this context that the numbers provided include all nucleated objects, including CTCs. Since their numbers were usually negligible compared to the numbers of leukocytes, they were not deducted from the total number of events, which was interpreted as the number of leukocytes.

Digital PCR

To lyse the cells, the membranes were transferred to 2 ml microcentrifuge tubes and incubated with 200 µl of lysis buffer (composition in Table S1) on a circular rotator for 1h at RT. DNA was then isolated using the QIAamp®DNAMicro Kit, following the manufacturer's instructions. Finally, the DNA was eluted in 20 µl nuclease-free water, and the concentration was measured using the Qubit™ 1X dsDNA High Sensitivity (HS) Assay Kit (Invitrogen). 18.5 µl of purified DNA was diluted in water 5-fold in a final volume of 92.5 µl, and 23 replicates of 4 µl of prediluted DNA (totaling in the whole

sample) were then mixed with Naica® PCR MIX 10X, Crystal Digital PCR® PIK3CA (E542-E545, E542K, E545K) Assay, and Crystal Universal Report 3.

All samples were then transferred onto the Ruby Chip of a Naica™ dPCR system (Stilla Technologies, Villejuif, France) that combines a droplet generator and thermal cycler. The following conditions were used for PCR reaction: NioProtocol_PIK3_3plex (95°C for 180s, then 60 cycles of 95°C for 15s, then 60°C for 30s, and a final step at 58°C for 300s). Water served as a technical negative control, and gDNA from MCF-7 cells was used as a positive control. The threshold for positive signals was set using two HD blood samples, treated equally to the spiking samples.

Supplementary Tables

Table S1: Lysis buffer recipe

Reagent	Manufacturer
6.6 mM Tris-acetate pH 7.5	Carl Roth, Karlsruhe, Germany
6.6 mM Magnesium-acetate	Carl Roth, Karlsruhe, Germany
33.3 mM Potassium-acetate	Carl Roth, Karlsruhe, Germany
0.43% Tween20	Carl Roth, Karlsruhe, Germany
0.43% IGEPAL CA-630	Carl Roth, Karlsruhe, Germany
0.10 unit of Proteinase K	800U/ml, New England Biolabs, Ipswich, MA, US
33.3 % PBS	Invitrogen, MA, US
Diluted in aqua dest	

Table S2: Summarized results of the comparative enrichment of CTCs from high-volume blood samples using either the bioaffinity CTC filtration membrane or the CellSearch® system.

Sample ID	Leukocyte number CTC-filter	Leukocyte number CellSearch®
**#1**	$1.91x10^5$	$3.97x10^3$
**#2**	$7.43x10^4$	$2.90x10^3$
**#3**	$4.77x10^5$	$1.81x10^4$
**#4**	$6.29x10^5$	$2.04x10^4$
**#5**	$1.10x10^6$	$2.60x10^4$
**#6**	$8.96x10^5$	$2.45x10^4$
**#7**	$2.91x10^5$	$4.82x10^4$
**#8**	$9.17x10^5$	$6.75x10^4$
**#9**	$5.01x10^5$	$4.59x10^4$
**#10**	$8.68x10^5$	$4.95x10^4$
**#11**	$9.33x10^5$	$4.18x10^3$
**#12**	$7.33x10^5$	$3.98x10^4$
**#13**	$6.74x10^5$	$1.64x10^4$
**#14**	$8.85x10^5$	$9.96x10^4$
**#15**	$8.49x10^5$	$5.11x10^3$
**#16**	$3.11x10^5$	$1.43x10^4$
**#17**	$3.27x10^5$	$6.24x10^4$
**#18**	$9.95x10^5$	$5.11x10^3$
**#19**	$2.40x10^5$	$1.80x10^5$
**#20**	$5.72x10^5$	$1.30x10^4$

Supplementary Figures

Figure S1

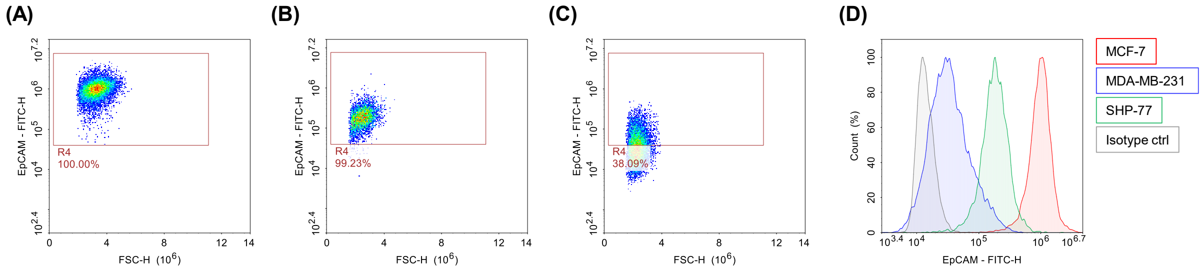

(E)

Sample ID	Average diameter [µm]
MCF7 CT orange	20.4
MCF7 unstained	17.2
MDA-MB-231 CT deep red	18.4
MDA-MB-231 unstained	16.3
SHP77 CT green	16.2
SHP77 unstained	12.7

Figure S1: (A) – (C) EpCAM expression on MCF-7, SHP-77, and MDA-MB-231 cell line cells assessed by FACS-analysis (D) Overlay of EpCAM signal intensity between all three cell lines and the Isotype control from MCF-7 cells. (E) Cell diameter with and without CellTracker (CT) labeling. All analyses were performed once.

Figure S2

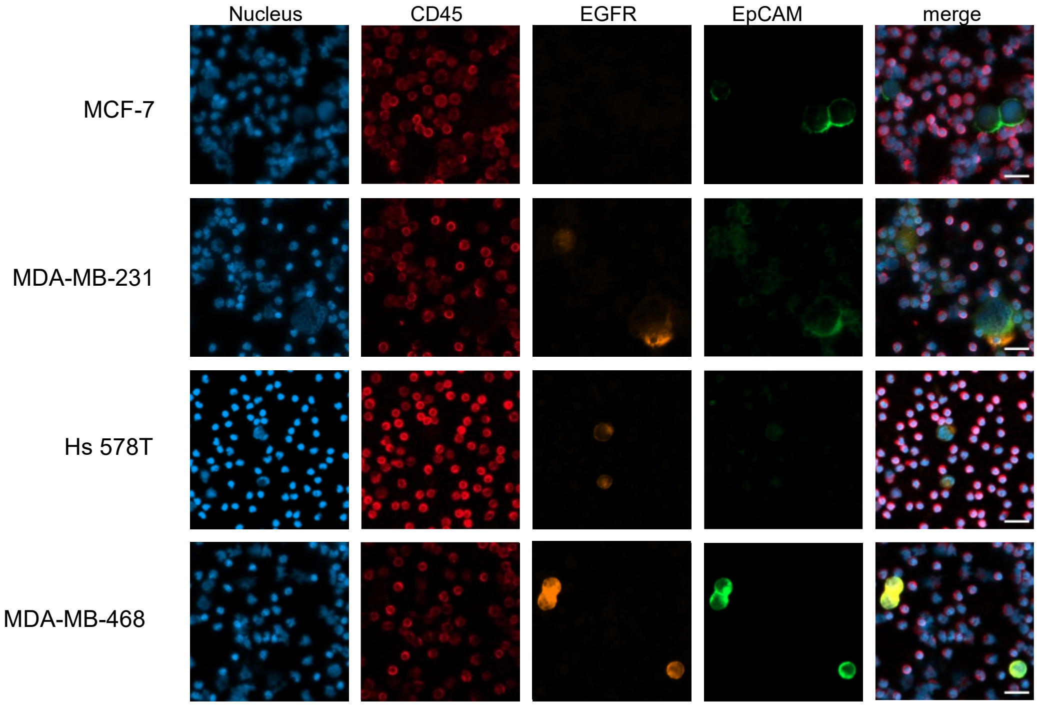

Figure S2: Representative images of MCF-7, MDA-MB-231, Hs 578T, and MDA-MB-468 cells spiked into HD blood, immunohistochemically stained against EGFR (orange) and EpCAM (green). Leukocytes were identified by anti-CD45 staining (red). Nuclei were counterstained with DAPI. The scale bar represents 20 µm.

Figure S3

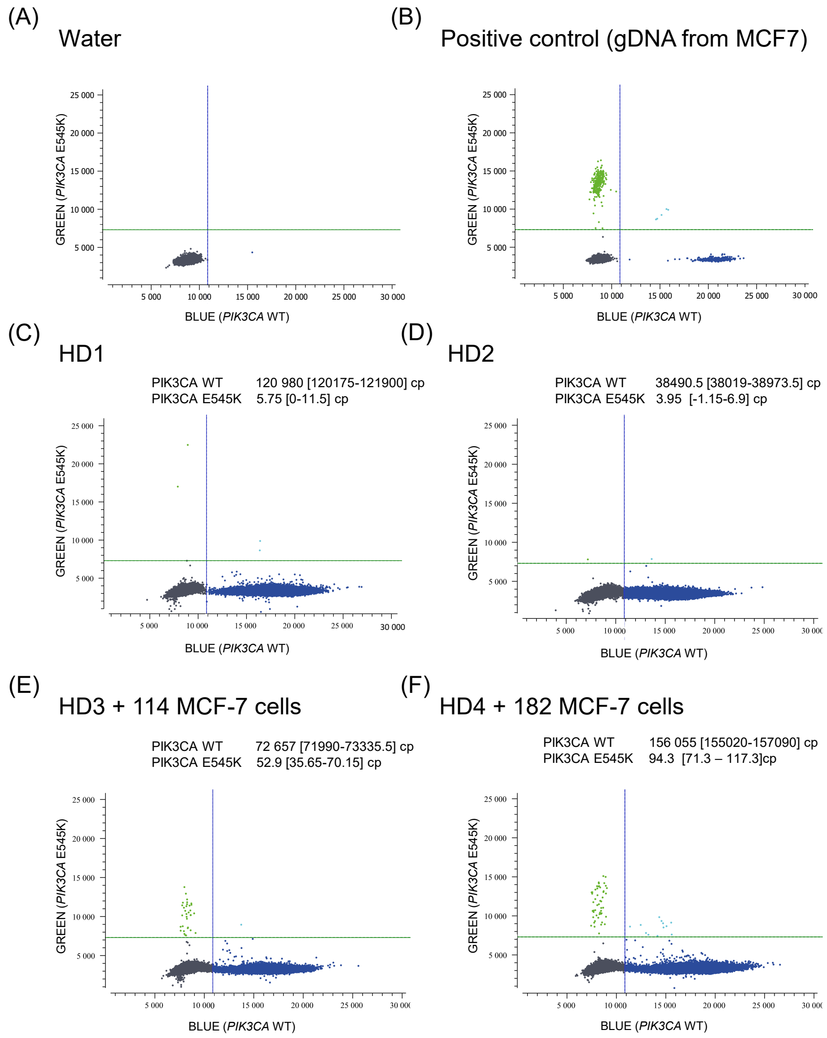

Figure S3: Detection of *PIK3CA* WT and E454K mutant copies in MCF-7 cells captured on bioaffinity membrane filters. (A) water control (B) gDNA of MCF-7 cells (positive control). (C), (D) Bulk analysis of leukocytes gDNA processed with the bioaffinity filtration membrane. (E), (F) Bulk analysis of gDNA of MCF-7 cells spiked into healthy donor blood and captured with the bioaffinity filtration membrane. The number of calculated copies per (whole) sample, as well as 95% confidence intervals, is provided for samples (C) – (F).

Supplementary References

1. Husseini H, Nielsen M, Pantel K, Wikman H, Riethdorf S, Werner R. Label Efficient Classification in Liquid Biopsy Data by Self-supervision. In: *Bildverarbeitung für die Medizin 2023*. Springer Vieweg, Wiesbaden; 2023:261-266. doi:10.1007/978-3-658-41657-7_58

2. Husseini-Wüsthoff H, Riethdorf S, Schneeweiss A, Trumpp A, Pantel K, Wikman H, Nielsen M, Werner R. Cluster-based human-in-the-loop strategy for improving machine learning-based circulating tumor cell detection in liquid biopsy. *PATTER*. 2025;6(6). doi:10.1016/j.patter.2025.101285

3. Schmidt U, Weigert M, Broaddus C, Myers G. Cell Detection with Star-Convex Polygons. In: Frangi AF, Schnabel JA, Davatzikos C, Alberola-López C, Fichtinger G, eds. *Medical Image Computing and Computer Assisted Intervention – MICCAI 2018*. Springer International Publishing; 2018:265-273. doi:10.1007/978-3-030-00934-2_30
